# Pulmonary Infection Due to *Trametes elegans* and *Peniophora incarnata*

**DOI:** 10.1093/ofid/ofaf575

**Published:** 2025-09-15

**Authors:** Hiroo Matsuo, Kensuke Fujiwara, Makoto Niki, Satoshi Kutsuna

**Affiliations:** Department of Infection Control and Prevention, Graduate School of Medicine, University of Osaka, Osaka, Japan; Department of Hematology, Hyogo Prefectural Amagasaki General Medical Center, Hyogo, Japan; Department of Infection Control and Prevention, Osaka Metropolitan University Hospital, Osaka, Japan; Department of Infection Control and Prevention, Graduate School of Medicine, University of Osaka, Osaka, Japan

**Keywords:** AIDS, climate change, fungal pneumonia, *Peniophora incarnata*, *Trametes elegans*

## Abstract

Recent environmental changes and advances in modern medicine—particularly the increasing use of immunosuppressive therapies and organ transplantation—have led to a growing number of infections caused by fungi that were previously not recognized as human pathogens. We report a case of pulmonary infection caused by *Trametes elegans* and *Peniophora incarnata* in a 27-year-old man with advanced acquired immunodeficiency syndrome who presented with multiple bilateral pulmonary nodules. Both fungi, which are rare environmental homobasidiomycetes, were identified using molecular methods. Thermal tolerance was demonstrated by *T. elegans*, supporting its ability to proliferate in human lungs, while *P. incarnata* showed limited growth at 35 °C. Initial treatment with liposomal amphotericin B was ineffective; however, the patient's condition improved following a switch to voriconazole. Climate change may increase the incidence of such opportunistic infections in individuals with compromised immunity.

In recent years, the landscape of fungal pathogens affecting humans has significantly changed. Global climate change, environmental disruptions, and advances in modern medicine, particularly the increasing use of immunosuppressive therapies and organ transplantation, have contributed to this shift [1, 2]. Consequently, fungi once regarded as nonpathogenic are now implicated in opportunistic infections. Notably, members of the phylum Basidiomycota, particularly Homobasidiomycetes, have emerged as unexpected pathogens in immunocompromised hosts [3].

Herein, we present a case of invasive pulmonary fungal infection in Japan caused by *Trametes elegans* and *Peniophora incarnata* that were both previously not recognized as human pathogens. Informed consent for publication was obtained from the patient and documented according to hospital regulations.

## CASE PRESENTATION

A 27-year-old Japanese man developed progressive weight loss (12 kg over 4 months), anorexia, dyspnea on exertion, and dry cough lasting 3 months. During a preoperative evaluation for anal fistula surgery, the patient was diagnosed with human immunodeficiency virus-1(HIV) infection and found to have multiple pulmonary nodules on imaging. Empirical liposomal amphotericin B (L-AmB) was initiated after filamentous fungi were detected in sputum cultures. Despite treatment, the patient's condition worsened, prompting bronchoscopy and molecular identification of 2 environmental fungi, *T. elegans* and *P. incarnata*. A switch to voriconazole and initiation of antiretroviral therapy resulted in clinical and radiologic improvement.

**Figure 1.**
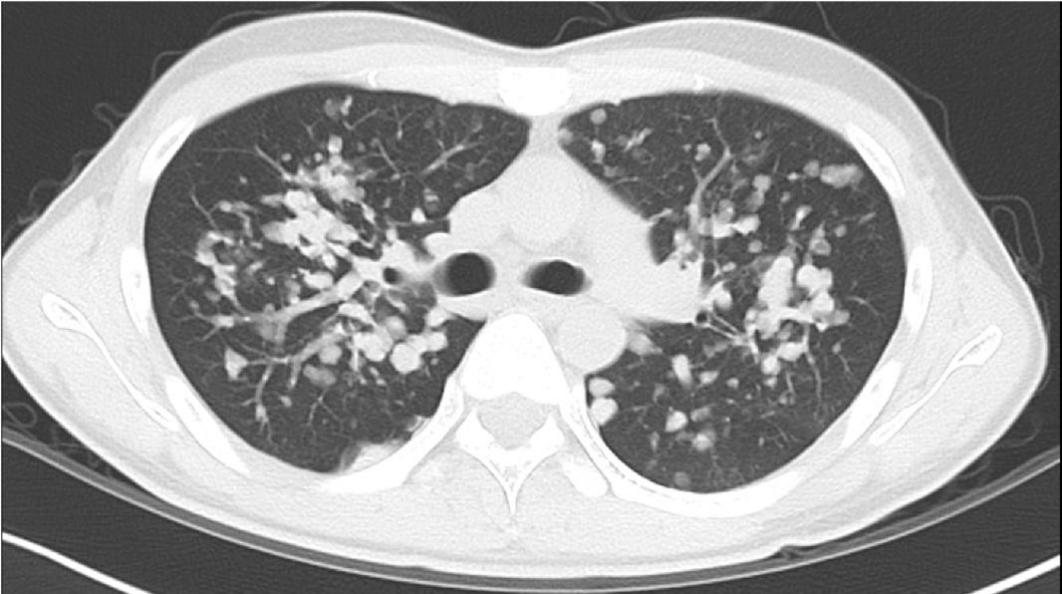
Chest tomography (CT) findings in a Japanese patient with acquired immunodeficiency syndrome (AIDS) and pulmonary infection caused by *Trametes elegans* and *Peniophora incarnata* in a study of emerging fungal infections. Multiple well-defined nodules are diffusely distributed throughout both lungs, with central predominance. Some lesions were also observed in the subpleural regions.

Physical examination showed no adventitious lung sounds but revealed cervical and inguinal lymphadenopathy and widespread molluscum contagiosum on the neck, back, buttocks, and lower extremities, consistent with severe immunodeficiency. The patient resided in an urban area with no known environmental exposures or outdoor hobbies. Laboratory results confirmed HIV infection with a viral load of 5.4 × 10^5^ copies/mL and a cluster of differentiation 4 (CD4) count of 28/μL. Serum β-D glucan was mildly elevated at 78.1 pg/dL. Both serum cryptococcal antigen and *Aspergillus* antigen tests were negative, thereby ruling out more common fungal pathogens. Chest computed tomography revealed numerous well-circumscribed nodules, predominantly in the central regions of both lungs (Figure 1).

**Figure 2.**
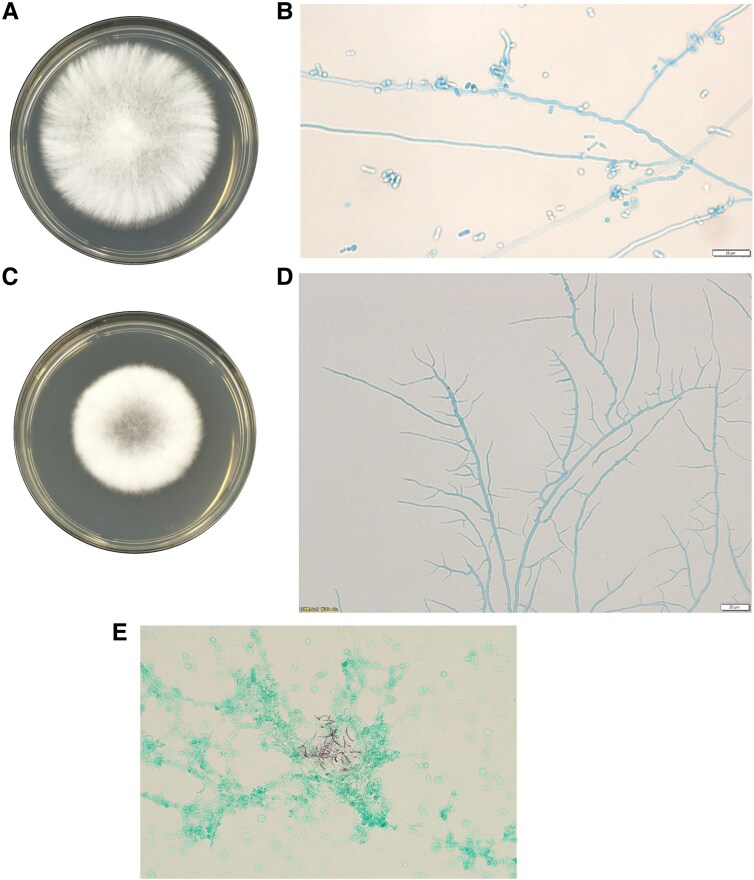
Imaging of fungal organisms isolated from airway secretions in a Japanese patient with acquired immunodeficiency syndrome (AIDS) and pulmonary infection caused by *Trametes elegans* and *Peniophora incarnata*. *A*, Colony morphology of *T. elegans* incubated at 28 °C for 7 d on potato dextrose agar (EIKEN). *B*, Microscopic morphology of *T. elegans* stained with lactophenol cotton blue (original magnification ×400). *C*, Colony morphology of *P. incarnata* incubated at 28 °C for 7 d on potato dextrose agar (EIKEN). *D*, Light microscopy of *P. incarnata* stained with lactophenol cotton blue, showing thin septate hyphae without observable clamp connections (original magnification ×400). *E*, Grocott methenamine silver stain of filamentous fungi observed in bronchoalveolar lavage fluid.

Gram staining of sputum revealed filamentous fungi. Cultures on potato dextrose agar incubated at 28 °C for 7 days yielded 2 distinct white colonies (Figure 2*A* and *C*). Although photomicrographs of the primary culture were not retained, the 2 isolates exhibited differences in surface texture and growth patterns, prompting us to submit both for molecular identification. In light of early clinical suspicion for fungal infection, fungal cultures were incubated for at least 7 days to allow for the detection of slow-growing species such as basidiomycetes. These samples were referred to the Osaka Metropolitan University for molecular identification, which revealed *T. elegans* and *P. incarnata*. DNA was extracted from the fungal isolates, and amplification of the internal transcribed spacer (ITS) and D1/D2 regions of the 28S rDNA was performed using PCR. Sequencing was performed using the BigDye™ Terminator v3.1 Cycle Sequencing Kit (Applied Biosystems) and analyzed on an Applied Biosystems 3130xl Genetic Analyzer. The resulting sequences were subjected to BLAST searches using the MycoBank database (https://www.mycobank.org/). For *T. elegans*, the ITS and D1/D2 sequences showed 99.8% and 99.7% similarity, respectively, with 100% coverage. For *P. incarnata*, the ITS region showed 99.8% similarity and 100% coverage. Although D1/D2 sequencing for *P. incarnata* yielded a top hit identified only at the genus level (*Peniophora* sp.), the ITS region supported species-level identification. Based on these results, we identified the isolates as *T. elegans* and *P. incarnata*. Microscopic examination of the isolate identified as *P. incarnata* revealed thin, hyaline, septate hyphae without clamp connections. Although clamp connections are a characteristic feature of homobasidiomycetes, they were not observed in this case, which may reflect environmental influences during culture, such as nutrient composition or temperature.

Given the findings suggestive of invasive fungal disease in the setting of advanced immunosuppression, we empirically initiated L-AmB (3 mg/kg/day) on Day X. Despite therapy, the patient's respiratory symptoms worsened, and repeat imaging revealed progression of the pulmonary lesions. On Day X + 12, L-AmB was discontinued owing to clinical deterioration, and bronchoscopy was performed on Day X + 15. Filamentous fungi were detected in the bronchoalveolar lavage fluid (Figure 2*E*), confirming an ongoing fungal infection.

Given the lack of clinical response and fungal identification, we initiated intravenous voriconazole (6 mg/kg every 12 hours) on Day X + 20. On Day X + 22, combination antiretroviral therapy (tenofovir alafenamide/emtricitabine and raltegravir) was administered. The patient subsequently showed improved respiratory symptoms and a reduced size and number of pulmonary nodules on follow-up imaging.

## DISCUSSION

*Trametes elegans* and *P. incarnata* are white-rot wood-decaying fungi that belong to the class homobasidiomycetes. These fungi are widespread in nature, but have rarely, if ever, been implicated in human diseases. *Peniophora incarnata* has a global distribution [4]. *Trametes elegans* is classically found in tropical and subtropical forests [5], including those in southern Japan. However, recent reports have documented its presence in regions of central Japan that were historically considered climatically unsuitable for its growth [6]. This apparent range expansion may be attributable to regional warming trends associated with global climate change [1], raising concern that such environmental shifts could increase human exposure to previously nonpathogenic fungal species.

Homobasidiomycetes, a subclass of Basidiomycota, are typically associated with wood decay in natural environments and rarely implicated in human diseases. However, there are occasional reports of invasive infections in certain species. For instance, *Schizophyllum commune* [7] has been reported in cases of sinusitis and pulmonary infections, whereas *Coprinopsis cinerea* [8] and *Bjerkandera adusta* [9] have been identified in a limited number of clinical settings, particularly among immunocompromised hosts. A broader group of filamentous basidiomycetes have been implicated in human disease and have been extensively reviewed in the literature [10–13]. These include species such as *S. commune*, *Hormographiella aspergillata*, *Ceriporia lacerata*, and *T. polyzona*. Our case contributes *T. elegans* and *P. incarnata* to this expanding list. Such infections are extremely rare, and these fungi are not traditionally considered human pathogens. This underscores the exceptional nature of the current case in which 2 homobasidiomycetes species were concurrently isolated from a patient with invasive pulmonary disease.

Importantly, the identification of homobasidiomycetes in clinical specimens poses diagnostic challenges. These organisms often require molecular methods for accurate identification because their morphological characteristics can be indistinct or overlap with those of other nonpathogenic environmental fungi. Standard clinical microbiology laboratories may lack the capacity to detect or classify these fungi, increasing the risk of misdiagnosis or delayed treatment [14, 15].

**Figure 3.**
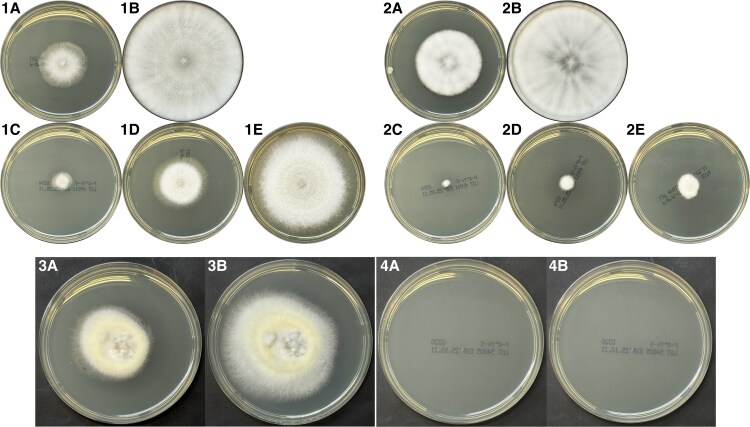
Temperature-dependent growth of *Trametes elegans* and *Peniophora incarnata*. Overview: On potato dextrose agar (EIKEN), *T. elegans* grew at 30 and 35 °C and, although more slowly, continued to expand at 37 °C. *Peniophora incarnata* formed colonies at 30 and 35 °C (weaker at 35 °C) but showed no visible growth at 37 °C. Comparability: Within each temperature set, the inoculum size was matched between species; images at 37 °C were obtained in follow-up experiments using different inoculum amounts from the 30/35 °C sets. Therefore, absolute colony sizes should not be compared across these temperature sets. Panel key (as labeled in the figure). Trametes elegans: 30 °C-day 4/8 (1A/1B); 35 °C-day 4/8/18 (1C/1D/1E); 37 °C-day 12/16 (3A/3B). Peniophora incarnata: 30 °C-day 4/8 (2A/2B); 35 °C-day 4/8/18 (2C/2D/2E); 37 °C-day 12/16 (4A/4B).

Fungal pathogenicity in humans is partially determined by the organism's ability to grow at body temperature. Most environmental fungi exhibit optimal germination at ambient temperatures (18–22 °C), whereas pathogens like *Aspergillus fumigatus* grow optimally at 37–42 °C [16]. Temperature-tolerance tests, including additional assays at 37 °C, were conducted on both isolates (Figure 3). *Trametes elegans* showed robust growth at 30 °C, slower but substantial growth at 35 °C, and continued colony expansion at 37 °C, reaching near-complete coverage by day 16. *Peniophora incarnata* showed robust growth at 30 °C, slower growth at 35 °C, and no visible colony formation at 37 °C, even after 16 days. The 37 °C growth images were obtained from follow-up experiments performed after the initial 30 and 35 °C assays, and inoculum sizes were not strictly identical between the experiments; therefore, direct comparison of absolute colony sizes should be interpreted with caution. These results indicate that *T. elegans* possesses thermotolerance compatible with human body temperature, supporting its potential to cause infection in immunocompromised hosts. Together with evidence of its recent geographic expansion, these findings reinforce our hypothesis that climate change may increase the incidence of such opportunistic infections. In the context of recent climatic shifts, these thermal tolerance characteristics reinforce its potential clinical relevance. Taken together, these findings support the hypothesis that *T. elegans* is the primary pathogen, although a contributory role of *P. incarnata* cannot be completely excluded.

Owing to the absence of standardized antifungal susceptibility testing protocols for homobasidiomycetes, we performed antifungal susceptibility testing using the Yeast-Like Fungus DP (DP “EIKEN”; Eiken Chemical Co., Ltd., Tokyo, Japan), a commercially available preformed drug plate. Testing was conducted according to the broth microdilution method recommended in CLSI document M38-A2 (CLSI, 2008). *Trametes elegans* yielded conidia that were tested successfully, whereas *P. incarnata* did not, precluding testing. *Trametes elegans* displayed paradoxical growth effects during antifungal testing. Nonetheless, the minimum inhibitory concentration of voriconazole was lower than that of L-AmB, which is consistent with the observed clinical improvement following the switch to voriconazole. However, this must be interpreted with caution, as antiretroviral therapy was initiated concurrently, potentially contributing to immune reconstitution and improved fungal clearance. Given the limited evidence regarding the treatment of filamentous basidiomycete infections, clinical guidance may be inferred from management of *S. commune*, the most frequently reported species. The European Confederation of Medical Mycology/the International Society for Human and Animal Mycology global guidelines recommend voriconazole as the preferred agent for treating rare mold infections, including basidiomycetes [17]. In our case, the patient's clinical response to voriconazole supports this approach, although further validation is required for newly implicated species such as *T. elegans* and *P. incarnata*.

Further research is needed to elucidate the virulence mechanisms, antifungal susceptibilities, and host interactions of emerging fungal pathogens such as *T. elegans* and *P. incarnata*. As the range of clinically relevant fungal species expands, new diagnostic and therapeutic strategies must be developed.

This study has some limitations. First, we did not obtain histopathologic confirmation of fungal elements. Nevertheless, the consistent detection of filamentous fungi in Gram-stained sputum and Grocott-stained bronchoalveolar lavage fluid, the concurrent isolation of *T. elegans* and *P. incarnata* from respiratory specimens, and the marked clinical and radiologic improvement after antifungal therapy strongly suggest that these fungi were the causative agents of infection. Second, the precise role of *P. incarnata* remains unclear. Although the temperature growth profiles suggest *T. elegans* as the primary pathogen, the possibility of a synergistic or sequential infection cannot be excluded. The simultaneous isolation of 2 white-rot wood-decaying fungi from respiratory specimens complicates the interpretation of their respective pathogenic roles. In nonhuman settings, cocolonization by multiple wood-decaying fungi has been shown to enhance wood degradation compared with single-species infection and to alter hyphal morphology and enzyme production [18, 19]. Moreover, in other fungal systems, the presence of dual species has been reported to facilitate tissue adhesion [20]. A similar phenomenon may have occurred in this case, although further investigation is needed to clarify the potential interactions between the 2 species in the human lung environment. Third, antifungal susceptibility testing could not be performed for *P. incarnata*, and no standardized testing protocol exists for *T. elegans*. Thus, while our findings and clinical responses suggest that voriconazole is more effective than L-AmB, definitive conclusions cannot be drawn. Prior reports on white-rot fungi have suggested that voriconazole may be more effective than L-AmB [9], and our case supports this notion, although further studies are required.

To the best of our knowledge, this is the first report of a pulmonary infection caused by either *T. elegans* or *P. incarnata*. Despite the increasing awareness of unusual fungal pathogens, these species have not been previously isolated from human respiratory specimens. Therefore, this case represents a novel clinical entity that extends the spectrum of fungal species with potential pathogenicity in immunocompromised individuals.

## CONCLUSION

This case highlights the need to reconsider the pathogenic potential of rare environmental fungi to cause severe infections in immunocompromised individuals. *Trametes elegans* and *P. incarnata* have not been previously reported as causative agents of pulmonary infections in humans. In advanced acquired immunodeficiency syndrome (AIDS), impaired mucocutaneous defenses and profound CD4 lymphopenia may facilitate the colonization and invasion of uncommon fungi.

Our findings emphasize the need for increased awareness of nontraditional fungal pathogens, especially because global climate change and ecological shifts may alter the epidemiology of fungal diseases. Clinicians should consider these organisms in the differential diagnosis of fungal pneumonia in immunocompromised patients, particularly when conventional pathogens are not identified and standard antifungal therapies fail.
